# Clinical and demographic characteristics of children and adolescents with acute vertigo symptoms: A cross-sectional study

**DOI:** 10.3906/sag-2010-228

**Published:** 2020-12-17

**Authors:** Arzu YILMAZ, Sema Nilay ABSEYİ

**Affiliations:** 1 Department of Child Neurology, Ankara Training and Research Hospital, Ankara Turkey; 2 Department of Pediatrics, Ankara Training and Research Hospital, Ankara Turkey

**Keywords:** Vertigo, child, adolescent, epilepsy

## Abstract

**Background/aim:**

Vertigo is one of the rarely diagnosed disorders during childhood due to insufficient description of the children regarding their experiences to the physicians. The clinical features of children and adolescents admitted by acute vertigo symptoms were investigated to elaborate the subject retrospectively.

**Materials and methods:**

Between January 2017–July 2019, records of cases admitted with acute vertigo complaints to pediatric neurology were retrospectively examined.

**Results:**

Of 761 patients, mean age was 13.8 years, 64% (n = 487) were women, 22.6% (n = 172) of which were children (1–11 years). A total of 37.3% of the cases (n = 284) had unknown etiology of acute vertigo symptoms, 39.6% (n = 301) had acute vertigo, and 23.1% (n = 176) were considered with no organicity problems but a group of the families stopped cooperating to the full extent in the study. Among all the patients, 25.6% (195/761) had paroxymal vertigo, 6.8% (52/761) had migraine-associated vertigo, 4.5% (34/761) had psychogenic vertigo, and 2.6% (20/761) had epileptic vertigo. Epileptic vertigo was significantly higher in younger children (mean age = 10.6, F(3) = 8874, P < .001), and the ratio of its occurence was also higher among children (60%, χ2 (3) = 20.347, P < .001).

**Conclusion:**

Vertigo complaints are 1.7 times more common among the girls. Epileptic vertigo is significantly higher among the children. Among younger children, it seems important to consider epilepsy when vertigo emerged.

## 1. Introduction

Vertigo is a feeling of self-motion when there is no physical self-motion, or is a feeling of distorted self-motion while the head moves in a normal way [1]. Vertigo is easily identified, but rarely diagnosed in children since it’s difficult for them to describe properly what they feel to physicians [2]. This is also why the recorded number of cases increases when the children with undetected vertigo grow older [3]. Vertigo is simply episodes of dizziness that occur suddenly and last for a few minutes. The seizures usually occur in children aged from 1 to 2 years after they start walking; they can also experience nausea and vomiting. During the seizure, the child is conscious, experinces a sudden feeling of shock whereby he/she feels frightened and doesn’t want to move until the end of the seizure. If the child is urged to walk during this period, he/she loses his/her balance. While young children cry in shock, older children try to describe the event [3].

In the evaluation of a patient presenting with childhood vertigo, whether the event is acute or recurrent, the time of onset and severity should be determined. Patients’ physical and neurological examinations, as well as audiological evaluations are required. Neurologic examination before and after the episode is considered normal so long as electroencephalogram (EEG) and magnetic resonance imaging (MRI) are normal.

In this study, the clinical characteristics of children and adolescents presented to the hospital by acute vertigo symptoms were investigated retrospectively, and the comparison of vertigo and other variables and also the children and adolescents was carried out.

## 2. Materials and methods

Children and adolescents, presented to the Department of Pediatric Neurology by vertigo symptoms between January 2017 and June 2019 at Ankara Training and Research Hospital were retrospectively examined.

The group composed of the patients aged from 1 to11 years was defined as children, while the group composed of the patients aged from 12 to 19 years are called the adolescent group. Based on their vertigo symptoms, cases were categorized in three groups: having unknown etiology of vertigo symptoms, diagnosed by acute vertigo, and those examined with regard to other disciplines, but their families stopped cooperating to the full extent in the study. Age, sex, medical examination findings (cardiological, neurological, ophtalmologic **,** audiological, psychiatric), imaging, and EEG results were obtained from electronical data files.

Acute vertigo was classified into four groups: benign paroxymal vertigo, migraine-associated vertigo, psychogenic vertigo, and epileptic vertigo based on International Classification of Headache Disorders Diagnosis (ICHD-3) [3]. The diagnosis of paroxymal vertigo was based on age, attacts’ characteristics, and normal neurological findings between attacks. The diagnosis of migraine-associated vertigo was made if the patient had headache following or accompanying vertigo attacks. The diagnosis of psychogenic vertigo was based on normal neurological and vestibular examination of the patients who described association of attacks with stressful situations supported by the child psychiatry examination. Epileptic vertigo was diagnosed for cases with vertigo complaints based on abnormal EEG (localized or generalized EEG changes) after vertigo attack, and the supporting clinical findings during these seizures.

Cases’ family history could not be evaluated because of the lack of data. No case had any drugs or trauma history according to the electronic record in retrespective analysis.

Ethics committee approval was received for this study.

### 2.1. Statistics

SPSS 21.0 (IBM Corp., Armonk, NY, USA) was used. Categorical variables were expressed in frequency (n) and percentage (%). The suitability of the continuous variables to normal distribution was tested by Kolmogorov–Simirnov test and expressed with arithmetic mean, standard deviation. ANOVA, Kruskal–Wallis and Pearson’s chi-squared tests were used. P < .05 was considered as significant for dual comparison, and P < .012 was accepted for more than two comparisons considering Bonferroni correction.

## 3. Results

The mean age of 761 patients admitted by vertigo symptoms was 13.8 years and the standard deviation was 3.2 years (min = 1 years, max = 19 years). A total of 64% (n = 487) of the cases were girls and 36% (n = 274) were boys.

While 22.6% (n = 172) of the cases were children (1–11 years), 77.4% (n = 589) were adolescents (12–19 years). Among the children, 53.5% (n= 92) of the cases were girls, while 67.1% (n=395) of the adolescent patients were girls.

Among 761 cases, 37.3% of them (n = 284) had unknown etiology of vertigo symptoms, 39.6% (n = 301) had vertigo, and 23.1% (n = 176) were examined with regard to other disciplines to exclude organicity, but the process remained incomplete due to withdrawal of their family. Three groups were similar in terms of age and sex distribution (P > .05, Table 1).

**Table 1:** Demographic data and clinical features of the cases with acute vertigo symptoms (n = 761).

	Total sample	Unknown	Acutevertigo	Consultated toother clinics	Statistics	
	n = 761	n = 284	n = 301	n = 176	F or χ2	P value
Age (years)a	13.8 (3.2)	13.6 (3.6)	13.9 (3.0)	13.9 (2.8)	.588	.556
Sex, n (%)					4.038	.133
Girls	487 (64.0)	171 (60.2)	205 (68.1)	111 (63.1)		
Boys	274 (36.0)	113 (39.8)	96 (31.9)	65 (36.9)		
Age group, n (%)					1.372	.504
Children	172 (22.6)	70 (24.6)	62 (20.6)	40 (22.7)		
Adolescent	589 (77.4)	214 (75.4)	239 (79.4)	136 (77.3)		
MRI, n (%)					21.680	.000
Normal	404 (53.1)	98 (34.5)	214 (71.1)	92 (52.3)		
Abnormal	86 (11.3)	10 (3.5)	36 (12.0)	40 (22.7)		
EEG, n (%)					22.317	.000
Normal	549 (72.1)	150 (52.8)	254 (84.4)	145 (82.4)		
Abnormal	20 (2.6)	0	20 (6.6)	0		

χ2: Pearson’s chi-square test

Based on MRI findings, consultation group had significantly higher abnormal MRI data (22.7%, χ2 (2) = 21.680, P < .001). Abnormal EEG findings, on the other hand, were significantly higher in children diagnosed with vertigo (6.6%, χ2 (2) = 22.317, P < .001) (Table 1).

In acute vertigo group (n = 301), 64.8% (195/301) had benign paroxymal vertigo, 17.3% (52/301) had migraine-associated vertigo, 11.3% (34/301) had psychogenic vertigo, and 6.6% (20/301) had epileptic vertigo.

The number of cases of epileptic vertigo was significantly higher in children both in younger age group [(mean age was 10.6, F(3) = 8.874, P < .001)] and the older age group [(60%, χ2 (3) = 20.347, P < .001)] (see Table 2).

**Table 2:** Acute vertigo types and their comparisons in terms of the demographic data of the vertigo group (n = 301)

	Vertigo (n = 301)					
	Bening paroxymal	Migraine- associated	Psychogenic	Epileptic	Statistics	
	n = 195	n = 52	n = 34	n = 20	F or χ2	P value
Age (years)a	14.1 (2.8)	14.3 (2.8)	13.7 (3.1)	10.6 (3.6)	8.874	.000
Sex, n (%)						
Girls	135 (69.2)	36 (69.2)	24 (70.6)	10 (50.0)	3.259	.353
Boys	60 (30.8)	16 (30.8)	10 (29.4)	10 (50.0)		
Age group, n (%)						
Children	35 (17.9)	9 (17.3)	6 (17.6)	12 (60.0)	20.347	.000
Adolescent	160 (82.1)	43 (82.7)	28 (82.4)	8 (40.0)		

χ2: Pearson’s chi-square test

## 4. Dıscussıon

In this study, 761 patients aged between 1–19 years who presented with vertigo were investigated. Regarding sex, it was found that the number of girls were 1.7 times more than the one of boys. In the study of Batu et al. [4], this rate was 1.1 for the girls. In terms of age group, almost half of the cases among children were girls. Two out of three adolescents had vertigo symptoms. In a population-based study examining vertigo frequency and risk factors among adolescents, conducted by Flippopulus et al. [5], among 1482 school children aged between 12 and 19 years, girls’ relative risk for vertigo was reported as 1.17 [1.10–1.25]. A retrospective study conducted with 147 adoloescents also showed that girls had higher rate of vertigo than boys [6]. Higher risk of vertigo for the female sex could be explained, as previously studies did, by the fact that othostatic intolerance with autonomic integrity [5,7,8] is lower in girls than boys; however, there is still no detailed epidemiological studies related to this.

Benign paroxysmal vertigo (BPV) is the most common cause of episodic vertigo in children aged two to six years [4]. It is characterized by short-term vertigo attack, nystagmus, and postural imbalance. During the seizure, the child may have fear, nausea, and vomiting; and if the patient was doing an activity, he/she stops doing it and needs to find comfort with his/her parents or in a specific setting. No hearing loss or tinnitus is observed. Attacks may be clustered, no complaints between the attacks is reported, and the data obtained from neurological examination is normal [4]. Although there is head trauma and a peripheral vestibular lesion like vestibular neuritis as underlying risk factor of benign paroxymal vertigo [9], in this study sample, BVP was detected in roughly 2/3 of the cases, and no specific trauma or lesion was found.

Migraine-related vertigo was the second most common type of vertigo in this study followed by psychogenic vertigo and epileptic vertigo. In a retrospective analysis of 100 children and adolescents diagnosed with vertigo, Batu et al. reported that benign paroxysmal vertigo rate as 39% [4]. Other studies also showed that more than half of adolescents with vertigo have headaches [10,11,12].

Psychogenic vertigo is another type of vertigo seen in adolescents. Of 25% of the children, and among the adolescents presenting with psychogenic vertigo, as their neurological examination and autological evaluation are normal, the reason of complaints may be psychogenic and these complaints increase under stress [13]. Erbek et al [14] reported 10% rate of psychogenic vertigo which is similar to the one we found in our study ( 11.3%). Another two studies, conducted by Batu et al. [4] and Gruber et al. [13], reported this rate as 21% and 22%, retrospectively. Psychogenic disorders like depression and anxiety were shown as the most frequent causes with a rate of 8.2% [15].

Epileptic vertigo is characterized by episodic vestibular symptoms deemed to result directly from focal, intermittent epileptic discharges [16]. Batu et al. reported epileptic vertigo rate as 15% [4]. Kluge et al. [16] reported epileptic vertigo in 73 cases (10.6%) in a cross-section of 687 samples. In our study, epileptic vertigo rate was 6.6% among the whole vertigo patients, and 60% in the pediatric age group. Tarnutzer et al. reported that children have 8.7 times higher risk in developing epileptic vertigo than adults [17]. These results might be interpreted as epileptic vertigo is likely to be a common diagnostic issue in pediatric populations, rather than concluding that younger brain produces more vertigo symptoms with epilepsy.

In this study, the number of patients having vestibular migraine could not be obtained because the data acquisition process from the patients who were consulted to the otolaryngorhinology department was left incomplete. Batu et al. reported vestibular migraine rate as 11% [4]. In a review evaluating 9 studies done with eight hundred patients, paroxysmal vertigo rate was reported as 18.7% and vestibular migraine rate as 17.6% [15].

## 5. Limitations

This study is a retrospective design and that data of approximately a quarter of case who were consultated to other disciplines to rule out other organic etiological factors did not completed due to their families’ withdrawal. Therefore, our findings cannot be generalized to all vertigo cases.

In conclusion, vertigo is 1.7 times more common in girls, and adolescent girls have a higher risk of having vertigo. The presence of epilepsy after vertigo is significantly higher in the pediatric age group. In the pediatric age group, it seems important to consider epilepsy too, when vertigo is diagnosed.
